# Mr. Knowles's Method of Preserving Leeches

**Published:** 1810-04

**Authors:** E. L. Knowles

**Affiliations:** Newmarket


### *To the Editors of the Medical and Physical Journal.*

Gentlemen,

I Lament exceedingly that the mortality amongst those valuable aquatic animals (leeches) is become of so great a consequence from a want of knowledge in the preservation, and improper treatment after suction, that renders it almost impossible to procure them even at an enormous price; it is obvious to every medical practitioner, that the utility of them in local inflammations is not to be exceeded, and in some cases, when their applications are immediately necessary, it becomes of the utmost consequence if they are not instantly obtained. As there are many parts where inflammation frequently attacks, that cupping glasses cannot be applied with any degree of advantage, it becomes a matter of importance, to ascertain a way of preserving them after being satiated with blood, and of cultivating them, that they may be procured more reasonable, and with greater facility calculated for the benefit of mankind.

Mr. Peck I observed, in your last Journal, has given some some instructions for the preservation of them, and treatment after suction, wherein he recommends keeping them in spring water. Is not spring water too cold and penetrating for this tender animal to exist in, as they are in general found in ditches and ponds, where the water is particularly soft? And when he comes to the mode of treatment after suction, he says, apply salt to the mouth of the leech, and it will disgorge part of the blood, and by a repetition it will entirely evacuate itself. I have frequently observed, that leeches by this mode of treatment, either will not evacuate themselves of the blood, or they are unable, and generally die. And he further mentions, to take particular care not to touch any other part of the leech with salt, except the mouth, or else it will produce blisters, and cause immediate death.

I believe there is no insensible membrane upon the mouth of the leech, I presume I may say granted; consequently, salt must have the same stimulating quality, and be productive of the same effects on the mouth as upon the body; and in my opinion, when blisters are excited upon the mouth, it becomes of more consequence than when on the body, as it renders the animal incapable of supporting life.

In a number of instances where I had occasion to apply them, the following mode of treatment has proved successful, and since I suggested this plan, I have not had a single leech die. Take a gentle hold of the leech and apply it to the part affected, and generally when she is full she will fall off of her own accord; then gently squeeze out the blood, (it will come out in a stream) then wash her clean in soft water, afterwards put her in a bottle, half filled with the same water; cover it with leather, and prick holes in it to admit air. When I have had a scarcity of them, I have frequently reapplied the same leeches in less than five minutes after the first operation, where necessity required, and they extracted as much blood the second time as on the first application, and without doing any injury to themselves, if judiciously managed.

As to Mr. P's other observations on this animal, as a barometer, they are very rational; but as experimental facts have convinced me that his plan is erroneous, that is, in the mode of treatment after suction, it induced me to take up my pen to communicate to your notice my observations and mode of treatment.

I am, &c.

*Newmarket, February* 9, 1810. E. L. KNOWLES.

